# Chloroaluminate Gel Electrolytes Prepared with Copolymers Based on Imidazolium Ionic Liquids and Deep Eutectic Solvent AlCl_3_:Urea

**DOI:** 10.3390/polym13071050

**Published:** 2021-03-27

**Authors:** Jesús L. Pablos, Pilar Tiemblo, Gary Ellis, Teresa Corrales

**Affiliations:** Instituto de Ciencia y Tecnología de Polímeros (ICTP-CSIC), Juan de la Cierva 3, 28006 Madrid, Spain; ptiemblo@ictp.csic.es (P.T.); gary@ictp.csic.es (G.E.)

**Keywords:** polymer gel electrolyte, chloroaluminate ionogel, imidazolium ionic liquid, aluminium secondary battery

## Abstract

Polymer gel electrolytes (PGEs) have been prepared with copolymers based on imidazolium ionic liquids and the deep eutectic mixture of AlCl_3_:urea (uralumina) as liquid electrolyte. The copolymers were synthesized by photopolymerization of vinylpirrolidone or methylmethacrylate with imidazolium bis (trifluoromethane sulfonyl) imide (TFSI) ionic liquid monomer and mixed in an increasing range of wt.% with uralumina. The rheology and electrochemical activity of PGEs were highly dependent on the molar ratio of charged groups and copolymer content. Structure of the PGEs was studied by FTIR and Raman spectroscopy and a correlation between interactions polymer/uralumina and changes in speciation of uralumina was established. Despite the low molecular weight of the copolymers, the resulting polymer electrolytes develop elastomeric character associated with the binding ionic species. Although there is room to improve the electrochemical activity, in this study these new gels provide sufficient electroactivity to make them feasible alternatives as electrolytes in secondary aluminum batteries.

## 1. Introduction

In recent years, the development of aluminum-based batteries is considered of great interest as a potential alternative to the well-established lithium ion battery [1]. Several motivations for the research on this metal can be highlighted, including its low density, the fact that it is the most abundant metal in the Earth’s crust, its recyclability, and that its energy density is higher than that of lithium. The current progress in non-aqueous aluminum batteries has been recently reviewed, including the chemistry of the electrolytes used (room temperature ionic liquids and deep eutectic solvents) [2,3]. The replacement of conventional liquid electrolytes by solid ones is a common endeavor to all metal secondary batteries, because the latter are much safer since they avoid leaks of toxic or corrosive liquids, and limit or eliminate dendritic growth of the reduced metal at the anode and the consequent short circuits.

In the literature dedicated to aluminum secondary batteries, only a few studies have report the use of polymers to prepare solid electrolytes instead of liquid ones. These include the in-situ polymerization of acrylamide in a deep eutectic solvent (DES) [4], and more recently, the dissolution of a commercial polymer (PEO) in uralumina U150 (AlCl_3_:Urea 1.5:1 in molar ratio), which is dimensionally stable as result of the strong interactions between the polymer and the DES [5].

It should be pointed out that the electrodeposition of Al in uralumina has been directly related to Al_2_Cl_7_^−^ and [AlCl_2_urea)_2_]+ [6], and that in the presence of an excess of AlCl_3_ in uralumina, highly interactive, strong Lewis acids are present; AlCl_3_ and Al_2_Cl_7_^-^. Thereby, the content of AlCl_3_ in the eutectic mixture plays an important role in the electroplating process. In the AlCl_3_:Urea mixtures with a 1.5:1 molar ratio, the mixture is completely ionic and the acidic species [Al_2_Cl_7_]^−^ and [AlCl_4_]^−^ are present together with cationic species of the type [AlCl_2_nUrea]^+^. The Urea-Al coordination takes place through the oxygen atom of urea [7,8,9,10], with further coordination through the nitrogen atom of the urea molecule [11].

Since the electrochemical activity of Al electrolytes has been observed to decrease in the presence of solvents with lone electron pairs, it should be expected that polymers bearing lone electron pairs, such as PEO, should decrease the ability to electrodeposit Al due to strong interactions between the electron deficient species in Al electrolytes and the lone pair of electrons present in the polymer. However, it has been demonstrated that the use of ultrahigh molecular weight PEO allows the preparation of PGEs using very low polymer weight fractions in the gel, whilst maintaining the ability to electrodeposit and strip aluminum [5]. In addition, other polymers that have a liquid state above 60 °C and the presence of oxygen groups in the backbone in common, should be expected to interact readily with uralumina and become either completely dissolved, with enough electrochemical activity to be considered as suitable polyelectrolytes. On the other hand, in the case of room temperature ionic liquids (RTIL) containing AlCl_3_/1-butyl-3-methylimidazolium chloride (BMIC) as the electrolyte in an Al-ion battery, it has been described that their physicochemical properties are significantly affected by the structural symmetry, cation-anion interactions, and strong hydrogen bonds formed in the chloroaluminate ionic liquids [6,12].

Here, the strategy developed takes advantage of the synthesis of copolymers by UV-irradiation, which is proposed as a very promising alternative for the preparation of new polymers that allows the design of the desired properties as a function of structure and ratio of monomers selected. The subsequent dissolution of these copolymers in the liquid electrolyte is undertaken, which is far more simple, reproducible, and scalable. In our previous work, free-standing ion gel electrolytes that consisted of crosslinked polymethacrylates with imidazolium pendant groups and RTILs as the liquid phase have been developed. These new self-standing ionic gels presented a combination of properties significantly higher than analogous liquid electrolytes [13]. Moreover, photopolymerization exhibits unique advantages, such as ultrafast reactions with temporal and spatial control, solvent-free systems, and ambient temperature reactions avoiding any thermal degradation.

The solubility of polymers in chloroaluminates requires both interacting chemical groups in the polymer structure and chain mobility and/or free volume to allow the diffusion of the liquid electrolyte into the polymer bulk. The study of solubility in U150 of commercial polymers of varied chemical structure and physicochemical characteristics indicates that polymers have a liquid state at the mixing temperature and the presence of oxygen groups in the polymer chain in common. Both these conditions are necessary but not sufficient, since PVP and PMMA contain interacting chemical groups that could allow the control of the crosslinking points in copolymers with DES, but are insoluble in U150, and do not swell either [14]. Because of the limited solvation ability of the DES to dissolve solid-state polymers with high glass transition temperatures, along with an upper temperature limit of around 70 °C (defined by the thermal stability of AlCl_3_:urea), it would be advantageous to design copolymers in order to obtain powdered materials with a glass transition temperature (Tg) ≤ 70 °C to promote a homogenous interaction between polymer and DES.

For this purpose, the previously synthesized imidazolium bis(trifluoromethane sulfonyl)imide (TFSI) ionic liquid monomer (IMMA) [15] has been used to obtain copolymers with methyl methacrylate (MMA) or 1-vinyl-2-pirrolidone (VP), P(MMA-co-IMMA) and P(VP-co-IMMA), respectively, adjusting the copolymer composition to ensure a glass transition temperature < 70 °C. The copolymers were mixed with U150 in increasing range of wt.%, to produce polymer electrolytes where the copolymer was completely dissolved and with increasing viscosity to obtain solid-like PGEs. The structure and properties of the polyelectrolytes have been studied to determine the interactions of polymers and chloroaluminates, which contribute to balance the rheology and electrochemistry in these gel electrolytes.

## 2. Materials and Methods

### 2.1. Materials

All compounds and solvents were commercially available and used as received: 2-bromoethanol (Aldrich, Steinheim, Germany, 95%), triethylamine (Sigma Aldrich, Munich, Germany, 99%), methacryloyl chloride (Aldrich, 97%), 1-butylimidazole (Aldrich, 98%), hydroquinone (Panreac, Barcelona, Spain, 99.5%), methyl methacrylate, 1-vinyl-2-pyrrolidone, bis(trifluoromethane)sulfonamide lithium salt (Aldrich, 99.95%), milliQ water, dichloromethane (Aldrich, 99.99%), hexane (Scharlau, Bavaria, Germany, 99.8%), Uralumina150 (U150) (Scionix Ltd. (London, UK)), and urea (Fischer Scientific, Madrid, Spain, 99.5%).

### 2.2. Preparation Procedure

P(MMA-co-IMMA) and P(VP-co-IMMA) copolymers were synthesized by the photo-induced free radical polymerization of methyl methacrylate (MMA) or 1-vinyl-2-pirrolidone (VP), with a polymerizable ionic liquid, 1-(2-Methacryloyloxy) ethyl-3-butylimidazolium Bis(trifluoromethane sulfonyl) imide) (IMMA), synthesized as described in previous work [16]. Furthermore, the homopolymers PMMA and PVP were synthesized in a similar manner. Table 1 shows the copolymers with different molar ratios prepared in this work.

A mixture of the corresponding monomer, methyl methacrylate or 1-vinyl-2-pyrrolidone, and IMMA along with the photo-initiator (Irgacure 651) was prepared in a glass vial and degassed by nitrogen bubbling and ultrasound for 10 min. Then the solution was injected into a circular teflon mold in an oxygen-free atmosphere and radical photopolymerization was performed using UV irradiation (365 nm) for 30 min at RT, then maintained in the dark for 48 h to enhance the molecular weight of the polymer. Subsequently, the copolymer was purified by dissolving in acetone (PMMA copolymers), or methanol (PVP copolymers), and poured into hexane for three times. The polymer was dried under vacuum at 55 °C for 48 h to give a solid product. Yield: 78%.

The copolymers were characterized by NMR (^1^H and ^13^C) and FTIR spectroscopy (Supplementary Information Figures S1 and S2). FTIR spectra of P(MMA-co-IMMA) show characteristic signals assigned to PMMA and the polyionic liquid: ν_s_(C=O) at 1725 cm^−1^, ν(C=N) imidazolium ring at 1561 cm^−1^, ν_s_(CH_3_(N)) δ_s_CH_3_(N)HCH at 1465–1422 cm^−1^, ν_as_(SO_2_ ) at 1347–1331 cm^−1^, ν_as_(CF_3_) at 1181 cm^−1^, ν_as_(SNS) at 1054 and 1131 cm^−1^, ν_s_(SO) at 1045 cm^−1^. Similarly, P(VP-co-IMMA) show characteristic signals assigned to PVP: ν_s_(C-H) around 2980 cm^−1^, ν(C=O) at 1660 cm^−1^, ν_s_(C-N-C) at 1493 cm^−1^, ν_s_(C-N) and δ_s_(CH_2_) around 1300–1280 cm^−1^, and those corresponding to the polyionic liquid already described.

Inclusion complexes (IC) of urea and polymers, PMMA, PVP, P(MMA-co-IMMA), and P(VP-co-IMMA), were prepared as reported in the literature [17,18]. Here, 300 mg of polymer was dissolved in 15 mL of dioxane with 2.5 g of Urea. The mixture was heated at 90 °C for 20 h and poured in 100 mL of hexane to obtain the urea inclusion complexes.

Polycationic Gel Electrolytes (PGEs). Polymers (between 5–20 wt.%) were added stepwise to uralumina inside a glovebox under argon atmosphere ([O_2_] < 1 ppm, [H_2_O] < 1 ppm). Uralumina was placed in a glass beaker on top of a heating plate and the polymer powder was slowly added while continuously stirring with a glass rod and the temperature was gradually increased to 70 °C and held for 25 min until the mixture became partially solid and completely homogeneous.

### 2.3. Characterisation

Nuclear magnetic resonance, ^1^H-NMR, and ^13^C-NMR spectra were recorded on a *Varian-Mercury* 400 MHz Nuclear Magnetic Resonance Spectrometer using hexadeuterated dimethyl sulfoxide (DMSO-d_6_) as the solvent, and chemical shifts were referenced to DMSO-d_6_ signals at 2.5 and 39.52 ppm, respectively, as the standard.

Attenuated Total Reflectance/FT-Infrared (ATR-FTIR) spectra of polymers were obtained using a Perkin Elmer BX-FTIR Spectrometer coupled with a MIRacle^TM^ATR accessory (PIKE Technologies, Inc., Madison, WI, USA) accumulating 32 scans at a resolution of 4 cm^−1^. Additionally, to obtain spectra of the polyelectrolytes and neat uralumina, samples were compressed between 2 mm thick ZnSe windows and sealed inside the glovebox and a FT-IR Perkin-Elmer Spectrum-One was employed in transmission mode, accumulating 10 scans at a resolution of 4 cm^−1^. *Raman spectroscopy* was undertaken using a Perkin Elmer System 2000 NIR FT-Raman system (PerkinElmer Ltd., Beaconsfield, UK), with a 1064 nm wavelength Nd^3+^:YAG laser at 500 mW power, accumulating 200 scans at 4 cm^−1^ resolution. The Raman samples were prepared by filling a Wilmad Precission Class A (300 MHz) NMR tube (Wilmad Lab Glass, Vineland, NJ, USA) with the electrolyte. All vibrational spectral data obtained were analyzed with Perkin Elmer Spectrum software.

Differential Scanning Calorimetry (DSC) was performed on a Mettler DSC-823e instrument previously calibrated with an indium standard (Tm = 155 °C, ΔHm = 25.75 Jg^−1^). Polymer samples (4 mg) were placed in aluminum capsules and the thermal history was erased by heating to 180 °C at 10 °C/min and maintaining the temperature for 2 min. The samples were then cooled to −60 °C and then the temperature was increased to 180 °C at 10 °C/min rate under a nitrogen atmosphere.

Rheological behavior. Because of the sensitivity to moisture of these polyelectrolytes, their rheology has been characterized in a qualitative way inside the glovebox. Two simple procedures were employed: The inverted tube test, very often used in gels [19], and additionally a simple stretching test. For the tube inversion test, 5 g of each PGE was introduced into a glass or vial, which was then turned over. The sample was observed with a video camera for time periods of several minutes. Depending on the tube inversion test outcome, the electrolytes may be considered as liquids if they flow as soon as the vial is reversed; while electrolytes that do not flow during the day are considered as solids. An intermediate behavior is shown for electrolytes that do not flow on inverting the tube, but creep down the tube walls within some tens of minutes to a few hours. The elasticity was evaluated taking advantage of their sticky properties, by a stretching test using a glass rod, and their performance recorded by video camera. The corresponding videos can be accessed via Table 2.

Electrochemistry. A lab-made cell fabricated from PET-g (glycol-modified polyethylene terephthalate) using a 3D printer was filled with the gel electrolyte, placed into a glass recipient, and sealed. The cell is able to resist contact with U150 without apparent degradation or swelling for more than 96 h. The lid of the glass recipient is wired, and each electrode connected prior to closing the isolating encapsulation. Subsequently, the whole setup was removed from the glovebox in order to evaluate the electrochemical properties using an Autolab PGSTAT 302 potentiostat/galvanostat controller (Metrohm AG, Herisau, Switzerland) as reported elsewhere [5]. Impedance measurements were carried out at an amplitude of 20 mV from 10^6^ to 10^3^ Hz, and the ionic conductivity was obtained from the equivalent circuit obtained after adjusting the Nyquist diagram. The same electrochemical cells were employed for the voltammetric measurements, using a third Al electrode as a pseudo-reference. Cyclic Voltammetry (CV) was measured between −1.5 V to 1.5 V versus Al/Al^3+^ at 20 mV s^−1^ for around 100 cycles. The steady-state current was reached after approximately 10 cycles.

## 3. Results and Discussion

### 3.1. Synthesis of Copolymers and Gels of U150/Copolymer

**Figure 1:**
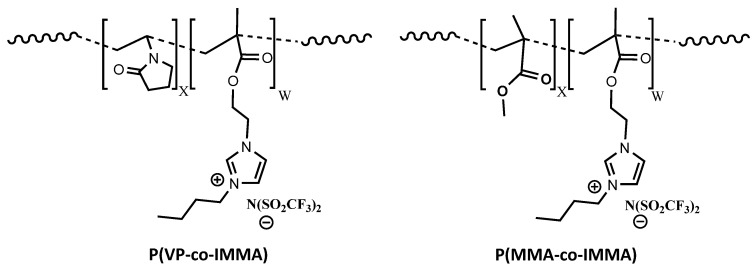
Structure of copolymers P(VP-co-IMMA) and P(MMA-co-IMMA).

The polymer gel electrolytes developed are based on copolymers synthesized via photopolymerization and dissolved in the deep eutectic mixture of U150. For that purpose, imidazolium bis (trifluoromethane sulfonyl) imide (TFSI) ionic liquid monomer (IMMA), was synthesized [16] and used to obtain copolymers with glass transition temperatures below 70 °C, to facilitate their dissolution in U150. In this respect, two sets of copolymers were prepared by photopolymerization, P(MMA-co-IMMA) and P(VP-co-IMMA), which exhibit tailored values of Tg. The samples were named using the monomer acronym (VP, MMA, or IMMA) with the molar ratio in the sub index of Table 1. The structure of the copolymers synthesized is provided in Figure 1.

The methodology used to prepare the polymer gel electrolytes can be described as a “solution” but also as “melt-mixing” as the polymers are only soluble in U150 above their glass transition temperature. A key factor in the polyelectrolyte preparation is the compatibilization of the polymer and the DES. Consequently, only copolymers with adequate values of Tg below 70 °C are used to guarantee a homogenous interaction between polymer and DES. The molar composition selected for vinylpirrolidone (VP) copolymers was 70:30, P(VP_70_-co-IMMA_30_), and for methylmethacrylate (MMA) copolymers two compositions were selected, 80:20 and 69:31, P(MMA_80_-co-IMMA_20_) and P(MMA_69_-co-IMMA_31_), respectively.

**Figure 2:**
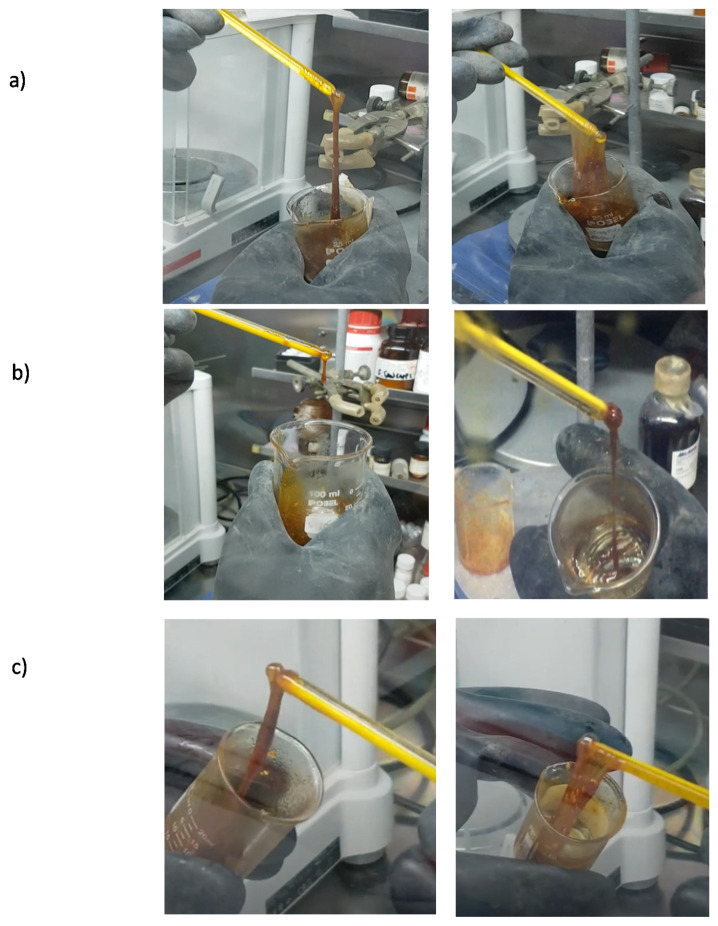
Rheology (**a**) P(MMA_80_-co-IMMA_20_)_5_/U150 (**left**), P(MMA_80_-co-IMMA_20_)_10_/U150 (**right**), (**b**) P(VP_70_-co-IMMA_30_)_5_/U150 (**left**), P(VP_70_-co-IMMA_30_)_10_/U150 (**right**), (**c**) P(MMA_69_-co-IMMA_31_)_5.9_/U150 (**left**), P(MMA_69_-co-IMMA_31_)_7.5_/U150 (**right**).

The selected copolymers in increasing range of wt.% were mixed with U150 in order to obtain polyelectrolytes where the polymer was completely dissolved and to determine the amount required to form a gel. P(MMA_80_-co-IMMA_20_) and P(MMA_69_-co-IMMA_31_) were used between 5 and 10 wt.%, and P(VP_70_-co-IMMA_30_) was used between 5 and 20 wt.%. The effect of the copolymer composition and the copolymer fraction in the blend could then be evaluated in terms of both the rheology and electrochemistry of the gels. Table 2 presents a summary of the electrolytes obtained, with the nomenclature employed including the composition of the PGEs and rheological properties, together with videos (as QR codes) showing their elastic properties and conductivity. Videos were recorded 24 h and 72 h after preparation to ensure that the blend formed is stable with time. The gels are sticky and elastic, as shown in the images of Figure 2 that were obtained from the videos. Uralumina is very sensitive to humidity and its manipulation requires a controlled atmosphere. Because of the viscosity increase and subsequent diffusivity decrease, the PGEs are far less sensitive to humidity but it is still not possible to study their rheology by routine techniques. Thus, to study the rheology of the gels, the inverted tube test was used, along with simple stretching tests that were video recorded.

The nomenclature chosen is the following: P(MMA_80_-co-IMMA_20_)_x_/U150, and P(MMA_69_-co-IMMA_31_)_x_/U150, where x refers to the copolymer weight fraction in the gel, seen in Table 2. The comparison of P(MMA_80_-co-IMMA_20_) versus P(MMA_69_-co-IMMA_31_), both at 7.5 wt.%, allows the determination of the influence of higher molar ratios of charged groups IMMA in the same amount of copolymer included in PGEs. Additionally, P(MMA_69_-co-IMMA_31_) at 5.9 wt.% compared to P(MMA_80_-co-IMMA_20_) at 7.5 wt.% allows the analysis of the behaviour of PGEs with same number of charged groups in the blend.

The analysis of the rheology and mechanical behavior of the PGEs obtained shows that the electrolytes with 5–10 wt.%, P(VP_70_-co-IMMA_30_)_5_/U150 and P(VP_70_-co-IMMA_30_)_10_/U150, behave as liquids of increasing viscosity, whereas P(VP_70_-co-IMMA_30_)_20_/U150 behaves as a solid with dimensional stability. In the case of those based on P(MMA_80_-co-IMMA_20_), elastomeric gel behavior is observed in P(MMA_80_-co-IMMA_20_)_10_/U150 whereas the rest of the solutions prepared with 5 and 7.5 wt.% of copolymer remained viscous liquids. Finally, the rheology of PGEs based on P(MMA_69_-co-IMMA_31_)/U150 does not change significantly and behaves as a viscous liquid even up to 7.5 wt.%, where a high content of imidazolium groups is present. Since P(MMA_80_-co-IMMA_20_)_10_/U150 (10 wt.% copolymer and 10.8 mmol of IMMA in 100gr PEG) behaves as an elastomeric gel, this seems to indicate that both high enough polymer and high enough IMMA content are necessary to prepare PGEs. Thus, it may be assumed that the interaction between uralumina and the copolymers may be a consequence of several factors; the presence of imidazolium groups that would promote the creation of crosslinking points in PGEs, apart from the presence of interacting chemical groups in the polymer structure that could lead to interaction between PMMA or PVP chains and urea, as previously described [12].

The elastic behavior of all the blends, and particularly the gel P(MMA_80_-co-IMMA_20_)_10_/U150, is very noteworthy. In fact, given the large deformation on strain of the gel, this material can be better described as elastomeric. Similar elastomeric behavior has been previously reported in gels prepared with uralumina and ultrahigh molecular weight poly(ethylene oxide) (UHMW PEO) [5], but it has not been detected in gels prepared with other polymers, which could be tentatively attributed to their molecular weight that was too low to develop elastic behavior. It must be pointed out that the presence of charged groups in these copolymers of polymerizable ionic liquids results in complicated and unreliable molecular weight characterization via gel permeation chromatography [20], and at present we do not have an accurate estimation of their molecular weight. However, it is likely that none of these copolymers is UHMW. This is why their elastic character is so remarkable and deserves further study.

**Figure 3:**
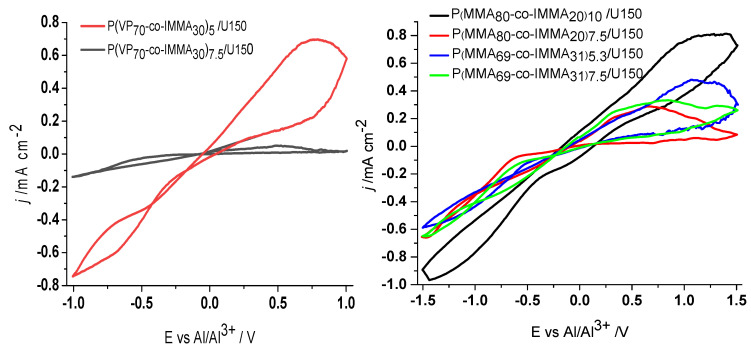
Cyclic voltammetry of PGEs P(MMA_80_-co-IMMA_20_)_7.5_/U150, P(MMA_80_-co-IMMA_20_)_10_/U150, P(MMA_69_-co-IMMA_31_)_5.9_/U150, P(MMA_69_-co-IMMA_31_)_7.5_/U150, P(VP_70_-co-IMMA_30_)_5_/U150 and P(VP_70_-co-IMMA_30_)_10_/U150.

In Figure 3, the cyclic voltammetry (CV) of polymer electrolytes are shown, and in Table 2, their ionic conductivity (σ) at 25 °C is presented in the last column. The analysis of data has been undertaken by comparison of PGEs with different content of the same copolymer, P(MMA_80_-co-IMMA_20_)_7.5_/U150 versus P(MMA_80_-co-IMMA_20_)_10_/U150, P(MMA_69_-co-IMMA_31_)_5.9_/U150 versus P(MMA_69_-co-IMMA_31_)_7.5_/U150, and P(VP_70_-co-IMMA_30_)_5_/U150 versus P(VP_70_-co-IMMA_30_)_10_/U150. In all cases, it was observed that, by increasing the polymer and therefore the IMMA molar ratio in PGEs, the viscosity increased to the detriment of the conductivity. The same trend is observed for P(MMA_80_-co-IMMA_20_)_7.5_/U150 versus P(MMA_69_-co-IMMA_31_)_7.5_/U150, with 7.5 wt.% of copolymer, where the conductivity was seen to decrease from 0.64 to 0.24 S cm^−1^, associated with the higher content of IMMA. Finally, σ values of P(VP_70_-co-IMMA_30_)_5_/U150 versus P(MMA_69_-co-IMMA_31_)_5.9_/U150 with similar wt.% of copolymer and IMMA groups have the same conductivity value of 0.44 S cm^−1^. The results obtained provide evidence for the significant influence of IMMA content on the activity of PGEs. It is concluded that interactions of imidazolium groups cannot be disregarded, in addition to those expected between carbonyl groups and U150.

A similar trend was observed in the CV curves showing the stripping/plating of Al, where the j of the gels decreased associated to the higher concentration of interacting groups and the increased viscosity, as shown in Figure 3. In all cases, a drop in current is seen compared to U150 (3.3 mA·cm^−2^; j value measured at 1 V) [5]. This decrease is similar to that found when adding 5 wt.% PEO of Mw 100,000, and much stronger than that found when adding 1 wt.% PEO of Mw 5,000,000. In addition, only one case can be considered a gel, P(MMA_80_-co-IMMA_20_)_10_/U150, while the rest are quite elastic liquids. It is noteworthy that this blend also exhibits the highest current, followed by P(MMA_69_-co-IMMA_31_)_5.9_/U150, P(MMA_69_-co-IMMA_31_)_7.5_/U150 and P(MMA_80_-co-IMMA_20_)_7.5_/U150, which are less viscous.

These results stress the importance of molecular weight in the preparation of gels with sufficient current to be feasible as gel electrolytes for secondary aluminium batteries. Unless gel electrolytes can be prepared with lower concentrations of copolycation, it is not possible to use them for this purpose. However, the tough, sticky, and elastic properties of P(MMA_80_-co-IMMA_20_)_10_/U150 are appealing since they lead to an electrolyte that is difficult to tear or break and that will efficiently wet the electrodes. On the other hand, it is possible to produce HMW polymethacrylates if the polymerization conditions employed are similar to living polymerization. Unlike other polymers like PCL, here there is a chance to produce HMW copolycations (Mw 10^6^) by radical polymerization [21,22,23] for the preparation of gels with very low polymer fractions (<5 wt.%), which may show solid character and high current.

### 3.2. Structure of the PGEs by FTIR and Raman Spectroscopy

FTIR spectroscopy has been employed to determine the interactions between uralumina and the different copolymers in PGEs, which have the presence of nitrogen and oxygen groups as side functionalities in common, more specifically, imidazolium, methacrylic ester, and lactam. The FTIR spectra of urea exhibits characteristic signals at 3432 and 3332 cm^−1^ corresponding to ν_as_ and ν_s_ (NH_2_), 1678 cm^−1^ assigned to ν_s_ (C=O), 1618 cm^−1^ attributed to δ_s_ (NH_2_), 1595 cm^−1^ assigned to δ_as_ (NH), and a peak at 1457 cm^−1^ corresponding to ν_as_ (C-N). Otherwise, the FTIR spectra of uralumina showed a slight shift in the ν_s_ and ν_as_ (NH_2_) signals and peaks assigned to ν_as_(C-N) and δ_s_ (NH_2_) when compared to urea, and the ν_s_ (C=O) peak shifted to 1655 cm^–1^. The observed shifts have been attributed to the formation of oxygen-to-metal coordinate bonds in uralumina [24,25], where the positively charged metal ion stabilizes the negative charge on the oxygen atom.

**Figure 4:**
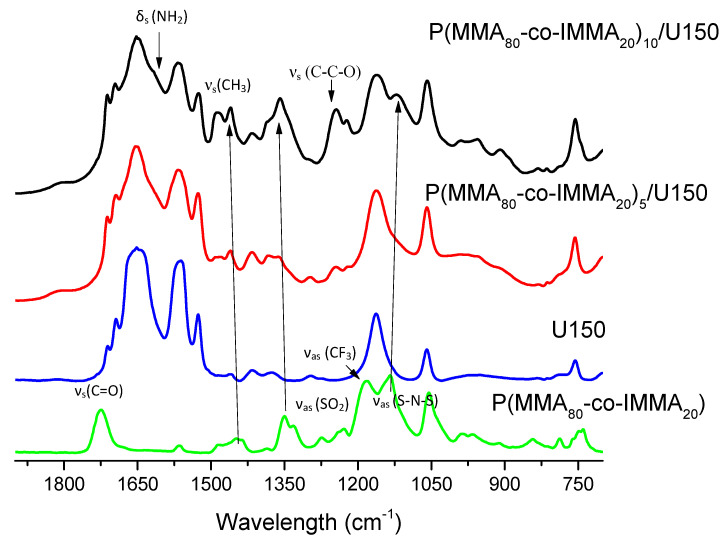
FTIR spectra of U150, P(MMA_80_-co-IMMA_20_), P(MMA_80_-co-IMMA_20_)_10_/U150 and P(MMA_80_-co-IMMA_20_)_5_/U150.

Since the viscosity of the blends increased through the mechanical mixing of copolymers with DES, it would be expected that the FTIR bands shift as a consequence of interactions between the components. In Figure 4, the FTIR spectra of PGEs based on P(MMA_80_-co-IMMA_20_)_x_/U150 at 5 and 10 wt.%, are compared to the spectra of copolymer and uralumina. The bands at 3150 and 3096 cm^−1^ corresponding to ν_s_(CH) and 2965 and 2940 cm^−1^ assigned to ν_as_(CH_2_) of polymer backbone were seen to increase with the content of copolymer and shifted very slightly in the polymer electrolytes with respect to the copolymer. This could be due to spatial rearrangements of the polymer chains due to the sum of interactions of polymeric chain and pendant imidazolium groups with uralumina.

The region 1100–1490 cm^−1^ contains bands due to different vibrational modes of the copolymer. Bands at 1245 cm^−1^ corresponding to ν_s_ (C-C-O), 1449 cm^−1^ (shifted to 1460 cm^−1^ in PGEs), and 1485 cm^−1^ assigned to ν_s_ (CH_3_) of PMMA are seen to increase with the concentration of the polymer. The strong intensity of bands corresponding to vibrational modes of the TFSI anion and their shift in PGEs with respect to the copolymer spectra, clearly suggest that the TFSI anion is involved in the interaction with U150. The band ν_as_ (SO_2_) at 1350 cm^−1^ shifts to 1358 cm^−1^, ν_as_ (CF_3_) at 1181 cm^−1^ disappears or shifts to 1220 cm^−1^, and ν_as_ (S-N-S) at 1136 cm^−1^ shifts to 1120 cm^−1^, as shown in Figure 4. The presence of the TFSI anion could lead to the formation of different structures of coordination with AlCl_3_, which would affect the aluminum speciation and therefore the electrodeposition. The results agree with those of other authors, where it was seen by Raman spectroscopy that uncoordinated TFSI anions decreased with increasing AlCl_3_ concentration in mixtures of ionic liquid with TFSI and AlCl_3_ [26,27].

The carbonyl band of the copolymer ν_s_(C=O) at 1724 cm^−1^ is not observed, albeit it is one of the most intense bands. This strongly suggests that all the carbonyl groups (also those belonging to the IMMA repeat unit) are involved in a very strong interaction with U150. As will be further detailed below, this is not surprising since PMMA is known to form inclusion compounds with urea [18].

Finally, a new band at around 1617 cm^−1^ appears to correlate with increasing concentration of polymer. This band could correspond with the δ_s_ (NH_2_) of weakly bound urea. In previous work, weakly bound urea formed in U150 polymer gels was already seen when preparing gels with the polyester PCL [14]. This occurs because the polymer displaces urea from the U150 complexed species, which could explain the decrease in conductivity of the gels as mentioned previously.

**Figure 5:**
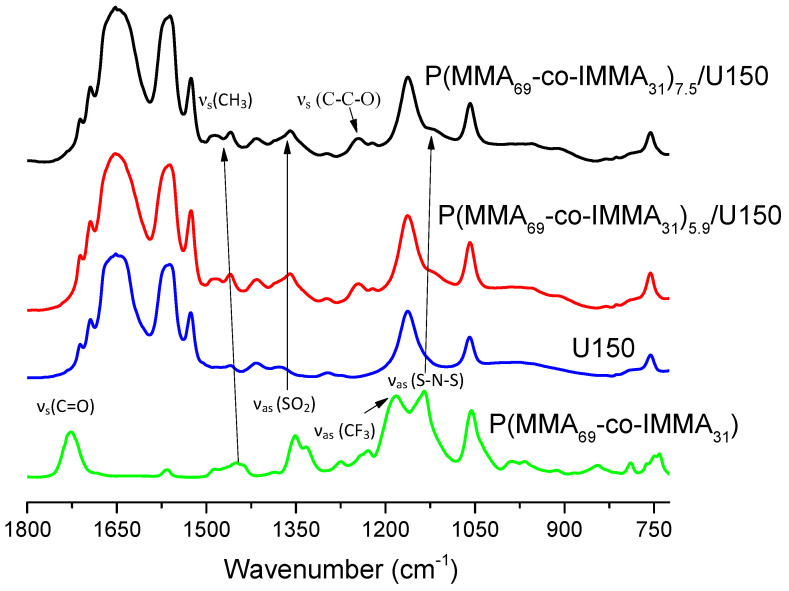
FTIR spectra of U150, P(MMA_69_-co-IMMA_31_), P(MMA_69_-co-IMMA31)_5.9_/U150, and P(MMA_69_-co-IMMA_31_)_7.5_/U150.

In a similar way, FTIR spectra of PGEs based on P(MMA_69_-co-IMMA_31_) showed the bands at 1449 and 1485 cm^−1^ corresponding to ν_s_(CH_3_) of PMMA and the absence of the carbonyl band of the copolymer ν_s_(C=O) at 1724 cm^−1^, as shown in Figure 5. However, in this case, the signal at 1619 cm^−1^ corresponding to δ_s_ (NH_2_) of pure urea is not well defined. The most significant difference is that changes assigned to vibrational modes in the TFSI anion occur to a lower extent than for P(MMA_80_-co-IMMA_20_)/U150, even with increasing wt.% of copolymer in PGEs. This could be related to the formation of ionic clusters by imidazolium groups in PGEs, which will be more favored as the content of IMMA in copolymer increases and, for instance, the interaction with U150 would be more hindered in P(MMA_69_-co-IMMA_31_)/U150 than P(MMA_80_-co-IMMA_20_)/U150. These results are in agreement with the rheology of PGEs based on P(MMA_69_-co-IMMA_31_)/U150, which behave as viscous liquids even up to 7.5 wt.% of copolymer, where the content of imidazolium groups is high and similar to P(MMA_80_-co-IMMA_20_)_10_/U150 that behaves as a gel.

Ionic clusters formation has been reported to be dependent on the anion, and a relation between number and size of cluster was established as well as the influence on their activity [28,29]. In previous work [16], it has been described that the fluorescence of imidazolium ionic liquid-based sensory membranes was fully attributable to imidazolium moieties. Membranes showed two emission maxima that could be associated with the emission from single molecules at short-wavelengths, whereas the long wavelength fluorescence component could be related to molecular aggregates of the ionic liquids. The addition of increasing concentration of Fe^3+^ resulted in decreasing the intensity of the shorter wavelength peak associated with the single molecules emission, while the peak at the longer wavelength corresponding to molecular aggregates remained practically constant, which is consistent with the higher availability of the imidazolium moieties as single molecules to form complexes with Fe^3+^.

**Figure 6:**
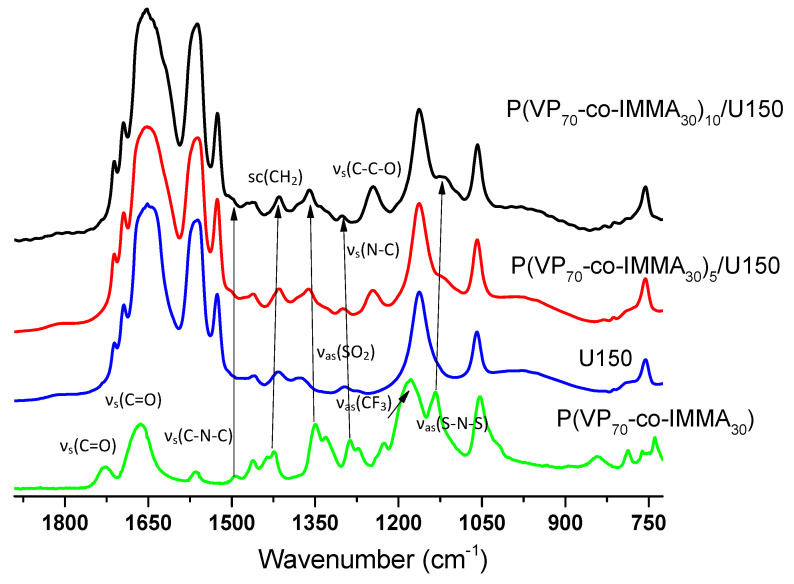
FTIR spectra of U150, P(VP_70_-co-IMMA_30_), P(MMA_69_-co-IMMA31)_5.9_/U150 and P(MMA_69_-co-IMMA_31_)_7.5_/U150.

In Figure 6, the FTIR spectra of PGEs based on P(VP_70_-co-IMMA_30_) at 5 and 10 wt. are compared to the spectra of the copolymer and uralumina. As previously observed for the PGEs described above, the peaks at 1725 cm^−1^ assigned to ν_s_(C=O) of IMMA and 1660 cm^−1^ corresponding to ν_s_(C=O) of amide in VP disappeared. The spectra contain bands arising from different vibrational modes of the copolymer; at 1493 cm^−1^ corresponding to ν_s_ (C-N-C), 1428 cm^−1^ to scissoring (CH_2_), 1292 cm^−1^ to ν_s_ (C-N), and 1245 cm^−1^ to ν_s_ (C-C-O), which are seen to increase with the concentration of the polymer. In general, a similar trend to P(MMA_69_-co-IMMA_31_)/U150 was observed, and small changes in the intensity of bands assigned to the TFSI anion were observed, even at 10 wt.% of copolymer incorporated into U150. This would be in agreement with the formation of ionic clusters when the content of IMMA in the copolymer is high, as was pointed out for P(MMA_69_-co-IMMA_31_)_x_/U150.

**Figure 7:**
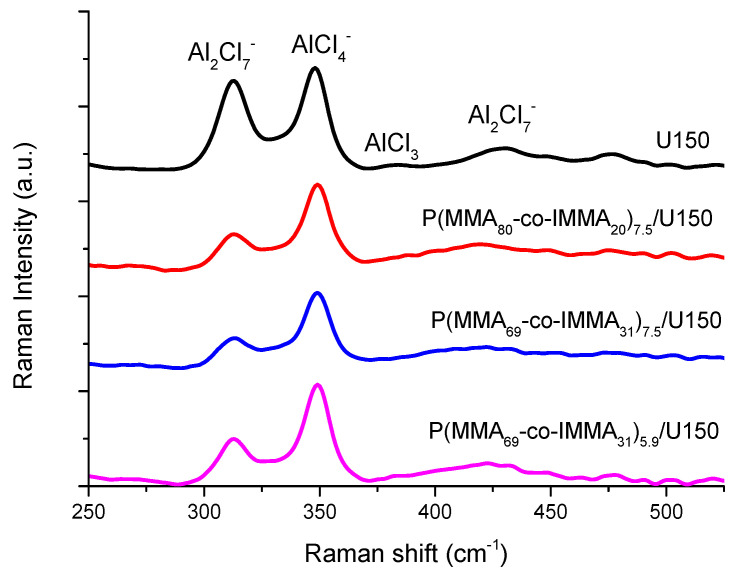
Raman spectra of U150, P(MMA_80_-co-IMMA_20_)_7.5_/U150, P(MMA_69_-co-IMMA_31_)_7.5_/U150, and P(MMA_69_-co-IMMA_31_)_5.9_/U150.

Raman spectroscopy. It is well known that the stripping/plating of Al is directly related to the presence of the ions Al_2_Cl_7_^−^ and AlCl_2_[urea]_2_^+^ [30]. It has been demonstrated that in polymer gels, the viscosity of the gel and its electroactivity are inversely correlated, since the interactions that promote the formation of gel reduce the concentration of Al_2_Cl_7_^−^ [5]. The fact that free urea is hinted in the FTIR spectrum would be an indication that the speciation of uralumina is changing, becoming less ionic and less acidic, which will have a deleterious effect on the electrodeposition of Al because of the decrease of Al_2_Cl_7_^−^ species and the increase of AlCl_4_^−^ and neutral species. The concentration of chloroaluminate anions in P(MMA_80_-co-IMMA_20_)_7.5_/U150, P(MMA_69_-co-IMMA_31_)_7.5_/U150, P(MMA_69_-co-IMMA_31_)_5.9_/U150 and neat U150 was characterized by Raman spectroscopy. In Figure 7, spectra are compared over the region between 200 and 650 cm^−1^. The Raman intensity of the bands assigned to Al_2_Cl_7_^−^ (312 cm^−1^) and AlCl_4_^−^ (348 cm^−1^) was normalized using the intensity of the 1057 cm^−1^ band corresponding to urea (associated with the symmetric CN stretch), and the results are collected in Table 3.

In the U150 spectrum, the characteristic signals at 312 and 433 cm^−1^ due to the dimeric Al_2_Cl_7_^−^ and the signal at 348 cm^−1^ corresponding to monomeric AlCl_4_^−^ anionic species [31,32,33] were observed, and correspond to that described by other authors for an AlCl_3_:urea ratio of 1.4:1 [34]. The spectra of copolymer-U150 electrolytes clearly show changes in the ratio between both species. In all the PGEs, a significant reduction of Al_2_Cl_7_^−^ band was observed with respect to its intensity in U150 as the copolymer content increases, clearly suggesting that Al_2_Cl_7_^−^ is not favored in the presence of the polymer chains. Comparison of the Raman spectra with the published work [34] suggest that P(MMA_80_-co-IMMA_20_)_7.5_/U150 and P(MMA_69_-co-IMMA_31_)_7.5_/U150 have an Al_2_Cl_7_^−^ concentration similar to that of uralumina with a urea:AlCl_3_ molar ratio of 1:1.2, and slightly higher for P(MMA_69_-co-IMMA_31_)_5.9_/U150. Moreover, AlCl_4_^−^ anions increase slightly in the PGEs. The results show that modification of the U150 composition could be different in each case, which also suggests that the type of interactions between the polymers and U150 is dependent on the type and content of the polymer. In addition, it cannot be disregarded that the presence of imidazolium TFSI structures in the mixtures of copolymers with AlCl_3_/urea could influence the relative proportion of chloroaluminate species, as previously reported in chloroaluminate ionic liquids [28].

Study of the inclusion complex in P(MMA_80_-co-IMMA_20_)/Urea and P(VP_70_-co-IMMA_30_)/Urea by FTIR, ^1^H-NMR, and DSC. In the FTIR spectra of PGEs, the disappearance of bands due to carbonyl group of PVP at 1650 cm^−1^ and PMMA and IMMA at 1727 cm^−1^ indicate that interaction between copolymers and U150 takes place. The ease of the urea molecule to form inclusion complexes (ICs) with a wide range of small molecules, aliphatic straight chain hydrocarbons, and linear polymers such us PMMA has been reported [17], as has the interaction between carbonyl groups of PVP and amine groups of urea [35,36,37]. Thus, it could be expected that a crosslinking reaction takes place directly in these complexes through urea molecules, or via the replacement of urea molecules by the polymer. Inclusion complexes of PMMA/Urea, P(MMA_80_-co-IMMA_20_)/Urea, PVP/Urea, and P(VP_70_-co-IMMA_30_)/Urea were prepared and studied by FTIR, ^1^H-NMR, and DSC. The main results of the analysis that suggest copolymer/urea interactions are summarized below. The FTIR spectra showed changes in the ratio of intensities of bands at 1458 cm^−1^ assigned to ν_as_ (C-N) and 1618 cm^−1^ and 1595 cm^−1^ corresponding to δ_s_ (NH_2_) and δ_as_ (NH) vibrations in comparison to neat urea. (Supplementary Information Figure S3). The ^1^H-NMR spectra (Supplementary Information Figures S4 and S5) showed the signal associated to the proton of the amine group of urea at 5.52 ppm, and a shift to 5.39 observed in the presence of PMMA or PVP and their corresponding copolymers. In the DSC thermogram of neat urea, an endothermic peak at 137 °C associated with the melting of urea tetragonal crystal structure was observed (Supplementary Information Figure S6). However, PMMA/Urea and P(MMA_80_-co-IMMA_20_)/Urea showed neither the Tg of PMMA nor the melting/recrystallization peaks of neat urea, and an endothermic peak appeared at around 110 °C that has been mainly associated with the disassociation of PMMA/U complexes, as well as the melting of small urea crystals. For the PVP/Urea and P(VP_69_-co-IMMA_31_)/Urea, an endothermic peak appeared at a slightly higher temperature, which could be related to a higher Urea/polymer mole ratio [17].

## 4. Conclusions

Copolymers based on vinylpirrolidone or methylmethacrylate and imidazolium bis (trifluoromethane sulfonyl) imide (TFSI) ionic liquid have been synthesized and mixed with U150 to obtain polymer gel electrolytes. Because of the limited ability of the DES to dissolve polymers, together with thermal stability of U150, copolymers were specifically designed with a glass transition temperature of around 70 °C, P(VP_70_-co-IMMA_30_), P(MMA_80_-co-IMMA_20_), and P(MMA_69_-co-IMMA_31_). By increasing both copolymer and IMMA molar ratio in PGEs, their viscosity increased to the detriment of the conductivity, which would evidence interactions of imidazolium groups in addition to those expected of the carbonyl groups with U150. Raman spectroscopy indicated that the speciation of uralumina changes, which is in line with a deleterious effect on the electrodeposition of Al, since the interactions that promote the formation of gel reduce the concentration of Al_2_Cl_7_^−^ species and the increase of AlCl_4_.

FTIR spectra indicated that all repeat units participate in the polymer/uralumina interaction (disappearance of C=O), and analysis of blends copolymer/urea by *FTIR, ^1^H-NMR,* and *DSC* confirmed that interaction takes place between carbonyl groups and amine groups of urea. In the same regard, the FTIR results highlight the fact that the TFSI anion is involved in the interaction with U150 (strong intensity and vibrational band shifts of the TFSI anion in PGEs with respect to the copolymer).

On the other hand, FTIR showed that changes in vibrational modes assigned to the TFSI anion take place to a lower degree in P(MMA_69_-co-IMMA_31_)/U150 and P(VP_69_-co-IMMA_31_)/U150 than for P(MMA_80_-co-IMMA_20_)/U150. This could be related to the formation of ionic clusters by imidazolium groups in the PGEs, which will be more favored as the content of IMMA in the copolymer increases and, for instance, the interaction with U150 would be more hindered. This is in agreement with the fact that all the polyelectrolytes behave as liquids of increasing viscosity, and only for P(MMA_80_-co-IMMA_20_)_10_/U150 is an elastomeric gel behavior observed. In that case, the elastic character is remarkable since, as far as we know, similar elastomeric behavior has been only found only in gels prepared with uralumina and ultrahigh molecular weight PEO.

The use of ultrahigh molecular weight polymers makes it possible to use very low weight fractions to obtain gels with U150, because of the effect of the polymer molecular weight on the rheology of polymer gels. Not all polymers can be obtained as UHMW; hence, when this is not possible, the only way to increase the modulus of a gel is by adding higher polymer weight fractions that, unfortunately, dramatically reduces the electrochemical activity. In addition to developing polymers with high molecular weight, this study proposes the preparation of tailor-made copolymers with charged groups as a novel strategy to induce the gel behavior, whilst maintaining sufficient electroactivity to make them feasible as electrolytes for secondary aluminum batteries.

## Figures and Tables

**Table 1:** Nomenclature and Tg values of the synthesized polymers determined by DSC.

Material	Tg (°C)
PVP	160
P(VP_97_-co-IMMA_3_)	155
P(VP_95_-co-IMMA_5_)	110
P(VP_90_-co-IMMA_10_)	89
P(VP_70_-co-IMMA_30_)	69
PMMA	105
P(MMA_80_-co-IMMA_20_)	70
P(MMA_77_-co-IMMA_23_)	63
P(MMA_69_-co-IMMA_31_)	40

**Table 2:** Nomenclature, composition, rheology, and conductivity of the materials.

Material	Polymer wt.%	10^3^ Moles IMMA in 100 g GPE	Aggregation State *		10^3^ σ (S cm^−1^)
U150	0	0	L	-	0.76
PVP/U150	5	0	I	-	-
P(VP_70_-co-IMMA_30_)_5_/U150	5	6.0	L	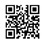	0.44
P(VP_70_-co-IMMA_30_)_10_/U150	10	9.5	L	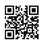	0.23
P(VP_70_-co-IMMA_30_)_20_/U150	20	12.7	S	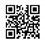	-
PMMA/U150	5	0	I	-	-
P(MMA_80_-co-IMMA_20_)_5_/U150	5	5.4	L	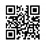	-
P(MMA_80_-co-IMMA_20_)_7.5_/U150	7.5	8.1	L	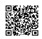	0.65
P(MMA_80_-co-IMMA_20_)_10_/U150	10	10.8	G	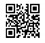	0.25
P(MMA_69_-co-IMMA_31_)_5.9_/U150	5.9	8.0	L	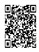	0.44
P(MMA_69_-co-IMMA_31_)_7.5_/U150	7.5	10.1	L	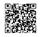	0.24

(*) Insoluble (I) Viscous liquid (L) Gel (G) and Solid (S).

**Table 3:** Variation in anion concentration estimated from integrated Raman bands corresponding to Al_2_Cl_7_^−^ (312 cm^−1^) and AlCl_4_^−^ (348 cm^−1^), and the urea band for normalization at 1057 cm^−1^. Column 2 is $\frac{I_{312}}{I_{1057}}\sim \frac{\left[Al_{2}Cl_{7}^{-}\right]}{\left[urea\right]}$, column 3 is $\frac{I_{348}}{I_{1057}}\sim \frac{\left[AlCl_{4}^{-}\right]}{\left[urea\right]}$, column 4 is $\frac{I_{312}}{I_{348}}\sim \frac{\left[Al_{2}Cl_{7}^{-}\right]}{\left[AlCl_{4}^{-}\right]}$, and column 5 represents the overall anion concentration related to the urea concentration.

PGEs	$\frac{I_{312}}{I_{1057}}$	$\frac{I_{348}}{I_{1057}}$	$\frac{I_{312}}{I_{348}}$	$\frac{I_{312}+I_{348}}{I_{1057}}$
U150	1.17	1.34	0.87	2.51
P(MMA_69_-co-IMMA_31_)_5.9_/U150	0.69	1.47	0.46	2.15
P(MMA_69_-co-IMMA_31_)_7.5_/U150	0.56	1.42	0.39	1.98
P(MMA_80_-co-IMMA_20_)_7.5_/U150	0.57	1.39	0.41	1.96

## Data Availability

The data presented in this study are available in the Supplementary Material.

## Supplementary Materials

The following are available online at https://www.mdpi.com/article/10.3390/polym13071050/s1, Figure S1: Characterization for P(VP_70_-co-IMMA_30_): A: NMR ^1^H, B: NMR ^13^C, Figure S2: Characterization for P(MMA80-co-IMMA20): A: NMR 1H, B: NMR 13C, Figure S3: FTIR spectra of (left) neat Urea, PMMA, P(MMA80-co-IMMA20), PMMA/Urea and P(MMA80-co-IMMA20)/Urea; (right) neat Urea, PVP, P(VP69-co-IMMA31), PVP/Urea and P(VP69-co-IMMA31)/Urea, Figure S4: NMR ^1^H of neat urea, PVP, P(VP70-co-IMMA30), PVP/Urea and P(VP70-co-IMMA30)/Urea, Figure S5: NMR ^1^H of neat urea, PMMA, P(MMA80-co-IMMA20), PMMA/Urea and P(MMA80-co-IMMA20)/Urea, Figure S6: DSC thermograms of neat Urea, PMMA/Urea, P(MMA80-co-IMMA20)/Urea, PVP/Urea and P(VP69-co-IMMA31)/Urea.
