# Supplementary material for: Chloroaluminate Gel Electrolytes Prepared with Copolymers Based on Imidazolium Ionic Liquids and Deep Eutectic Solvent AlCl3:Urea
Source: Polymers (Basel). 2021 Mar 27;13(7):1050. doi: 10.3390/polym13071050 (PMC8037023; doi:10.3390/polym13071050)

Chloroaluminate gel electrolytes prepared with copolymers based on imidazolium ionic liquids and deep eutectic solvent $AlCl_3$:urea

Supplementary Materials

Figure S1. Characterization for P(VP_{70}-co-$IMMA_{30}$): A: NMR ^1H, B: NMR ^{13}C

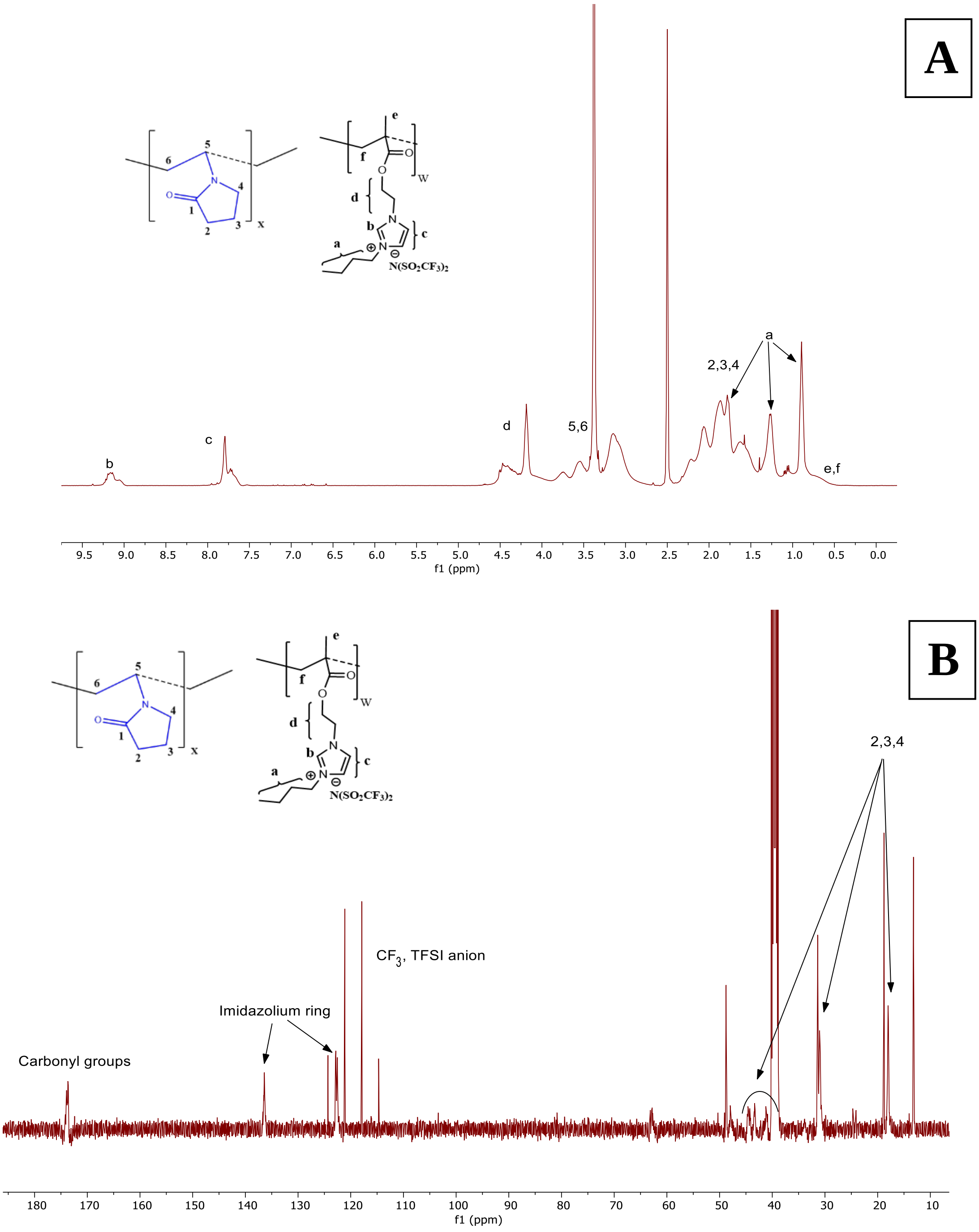

Figure S2. Characterization for P(MMA80-co-IMMA20): A: NMR ^{1}H, B: NMR ^{13}C

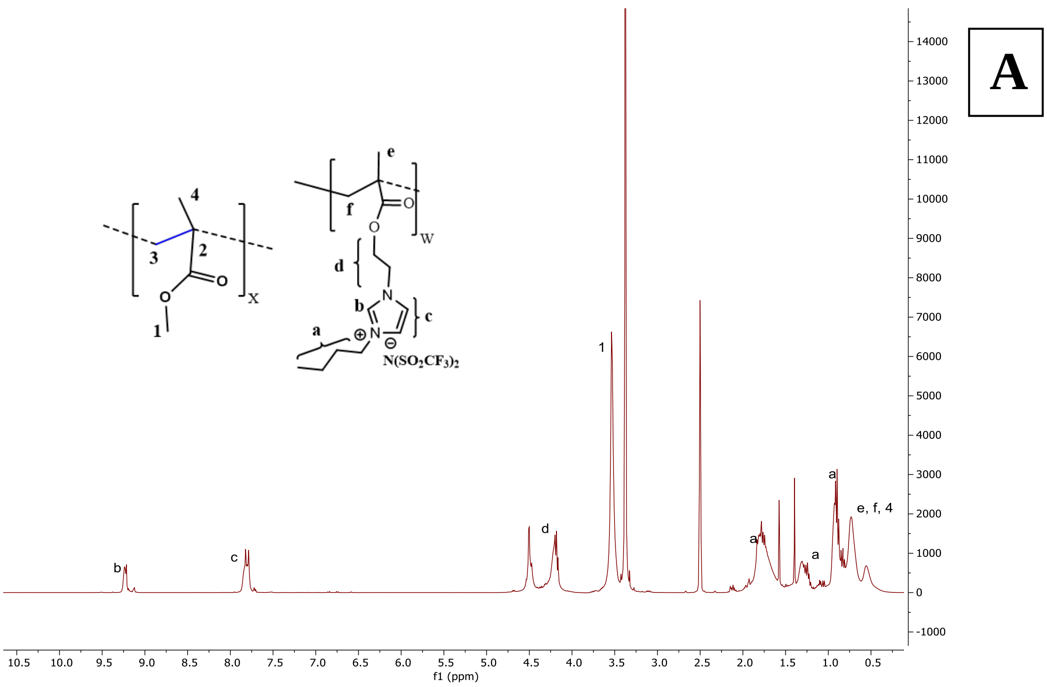

C:/Users/music/OneDrive/Escritorio/WS_FTP/JLR-MMAPolicR2/11/fid

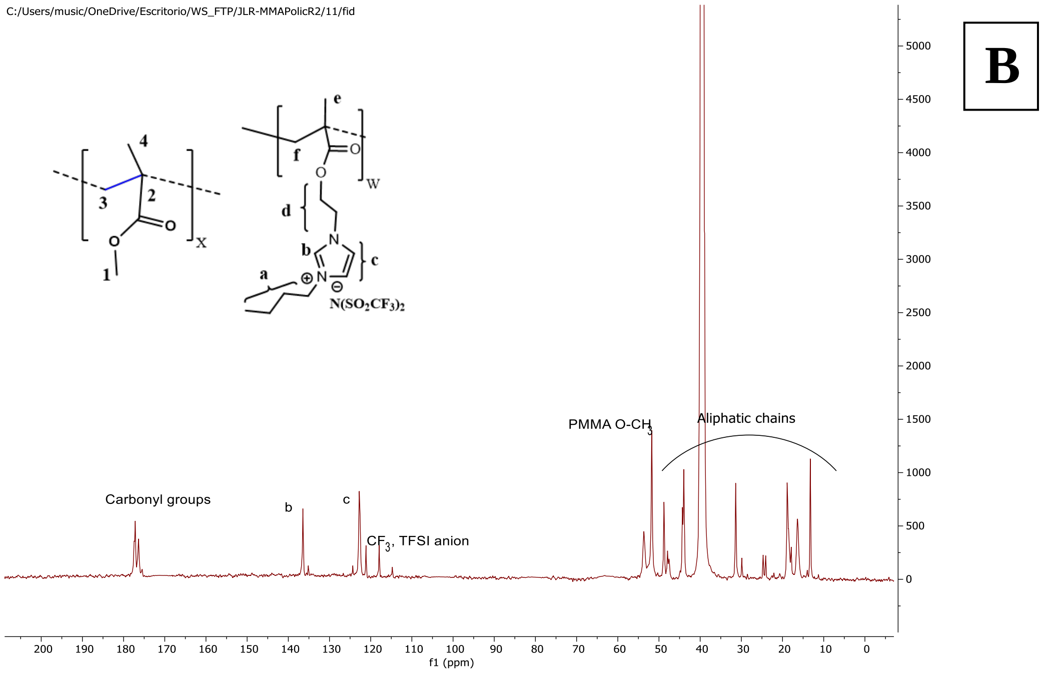

Figure S3. FTIR spectra of (left) neat Urea, PMMA, P(MMA$_{80}$-co-IMMA$_{20}$), PMMA/Urea and P(MMA$_{80}$-co-IMMA$_{20}$)/Urea; (right) neat Urea, PVP, P(VP$_{69}$-co-IMMA$_{31}$), PVP/Urea and P(VP$_{69}$-co-IMMA$_{31}$)/Urea.

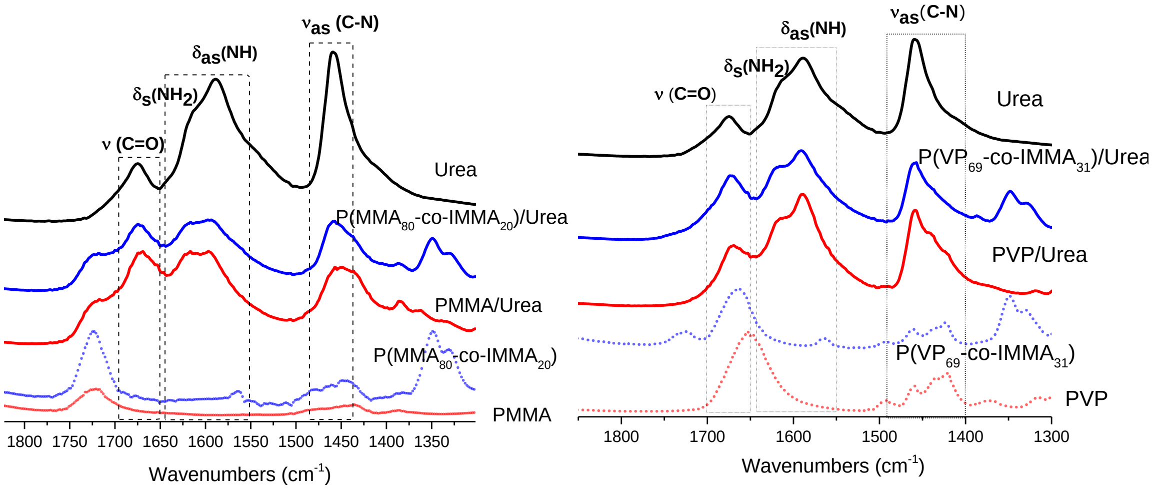

Figure S4. NMR ^{1}H of neat urea, PVP, P(VP$_{70}$-co-IMMA$_{30}$), PVP/Urea and P(VP$_{70}$-co-IMMA$_{30}$)/Urea.

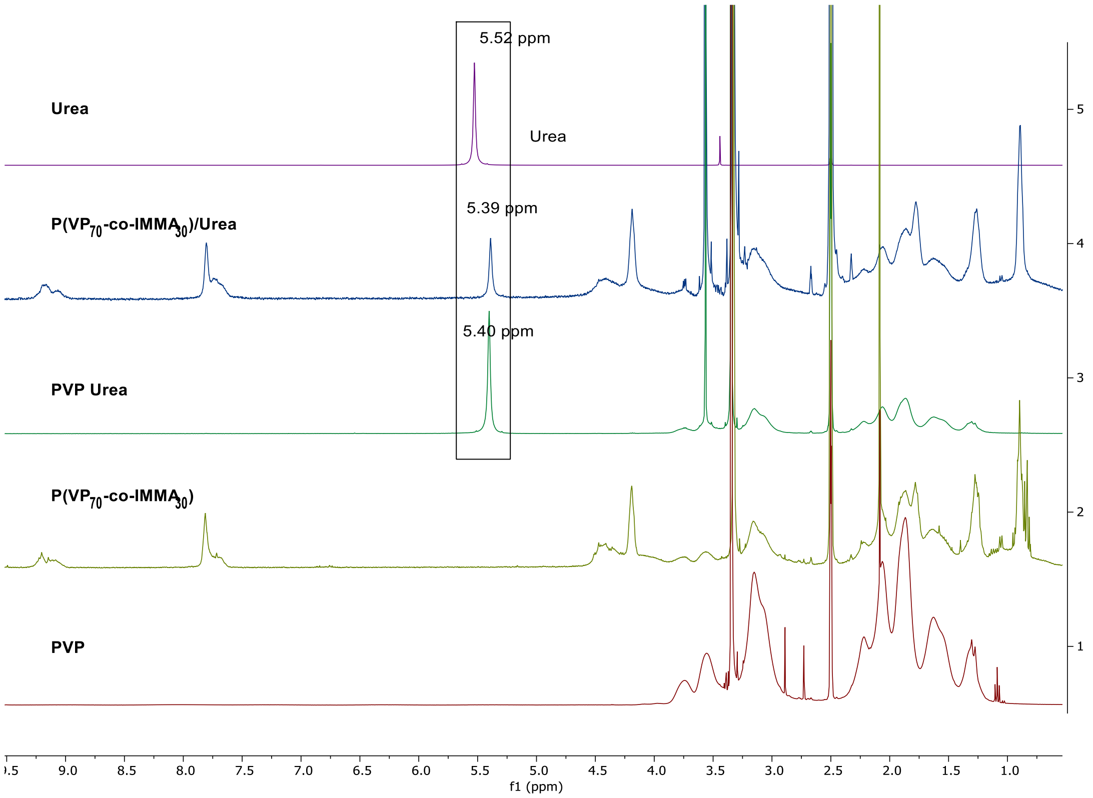

Figure S5. NMR ^1H of neat urea, PMMA, P(MMA_{80}-co-$IMMA_{20}$), PMMA/Urea and P(MMA_{80}-co-$IMMA_{20}$)/Urea.

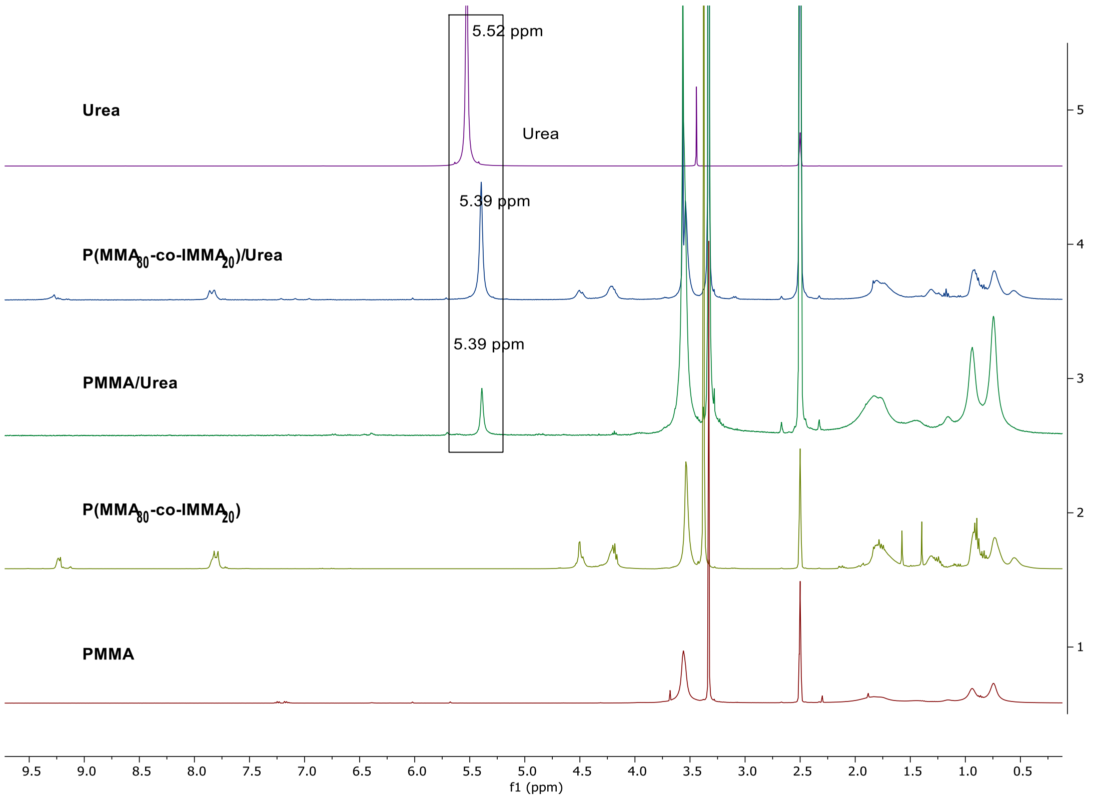

Figure S6. DSC thermograms of neat Urea, PMMA/Urea, P(MMA_{80}-co-$IMMA_{20}$)/Urea, PVP/Urea and P(VP_{69}-co-$IMMA_{31}$)/Urea.

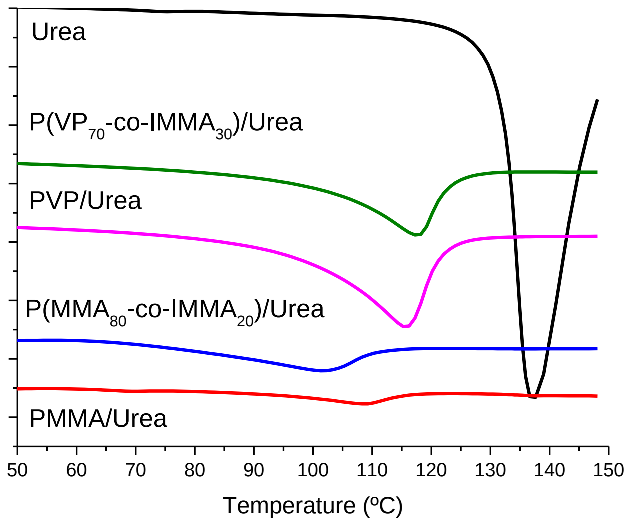

Supplement: Supplementary file 1 [file polymers-13-01050-s001.pdf]
